# HIV stigma in the teaching hospitals in Sana’a, Yemen: a conflict and low-resource setting

**DOI:** 10.1186/s12889-021-11845-y

**Published:** 2021-10-05

**Authors:** Bothaina Ahmed Attal, Kowthar Mohammed Al-Rowaishan, Alaa Abdulbaset Akeel, Fawziah Kassim AlAmmar

**Affiliations:** 1 Faculty of Medicine and Health Sciences, Sana’a University, The Sixty St., Sana’a, Yemen; 2 Affiliated researcher at the Centre for Business Research, Cambridge Judge Business School, Cambridge, UK; 3 Al-Thawra Hospital, Bab Al-Yemen, Sana’a, Yemen; 4 Relief International, Ali Kabus St, Sana’a, Yemen; 5 Educational and Psychological Sciences Department, Faculty of Education, Art and Applied Sciences, Amran University, Amran, Yemen

**Keywords:** HIV stigma, Conflict, Health care, Low HIV-prevalence, Contextual factors, Yemen

## Abstract

**Background:**

HIV stigma undermines a person’s wellbeing and quality of life and hinders HIV control efforts. This study examined the extent and drivers of HIV stigma in the teaching hospitals in Sana’a City, Yemen. The country has low HIV prevalence (4000 (2000-11,000) per 100,000) and limited HIV control funds, worsened by a long conflict and an economic crisis.

**Methods:**

We conducted a cross-sectional study of 320 Yemeni health professionals in all the four teaching hospitals in Sana’a City. Data were collected anonymously, using an adapted self-completed Arabic version of the Health Policy Project HIV Stigma tool. The questionnaire covered the respondents’ background, the stigmatising practices, and potential personal and professional drivers of stigma.

**Results:**

The majority of the participants were: females (68%), 20–39 years old (85%), nurses (84%), and holding a nursing diploma (69%) or a bachelor’s degree (27%). None of the hospitals had institutional policies against HIV stigma, and 93% of the participants believed the current infection control measures were inadequate. Less than half of the participants provided care for people living with HIV (PLHIV) (45%), had received HIV training (33%), and were confident that their HIV knowledge was adequate (23%). The majority indicated a preference to test patients for HIV prior to surgical procedures (77%) and disclose positive HIV results to others (99%) without prior knowledge or consent. All the participants had exhibited a form of HIV-related stigmatization, such as avoiding physical contact with PLHIV (87%) or wearing gloves throughout the consultation (96.5%). These practices were significantly correlated with the fear of infection, high perceived risk of infection, and poor work environment (*p* < 0.05).

**Conclusion:**

PLHIV face widespread stigmatizing behaviour in the teaching hospitals in Sana’a City, consistent with the higher level of stigma in low HIV prevalence countries and its links to the fear of infection, poor HIV knowledge, and limited funding for HIV control. Stigma reduction interventions are required at institutional and individual levels. In addition, anti-discrimination policies and structural adjustments are needed, in combination with training on HIV and universal precautions, and action to tackle negative attitudes towards PLHIV and key populations.

**Supplementary Information:**

The online version contains supplementary material available at 10.1186/s12889-021-11845-y.

## Background

Stigma is a social and cultural phenomenon in which an individual possessing a devalued characteristic, such as HIV infection, is discredited by others [1]. Identified by Link and Phelan, labelling, stereotyping, separation, status loss, and discrimination as various forms of stigma [2], and are considered a violation of human rights when targeted at people living with HIV (PLHIV) [3–6]. PLHIV experience the mechanisms of internalised, anticipated, and enacted HIV stigma [7]. Internalised stigma denotes the endorsement of negative beliefs, views and feelings of oneself, whereas anticipated stigma is awareness of negative social perceptions towards HIV, and the expectation that PLHIV will experience prejudice. Enacted stigma refers to the discrimination experienced by PLHIV [7]. This study focuses on the mechanism of enacted stigma, which refers to the discrimination experienced by PLHIV.

HIV stigma is considered a major catalyst of the HIV epidemic, and is reported all over the world in communities, work-spaces and other settings [8, 9]. Although health care contexts should be “safe and protective” spaces, PLHIV are frequently subjected to refusal of care, irrational use of infection control measures by care providers [10–12], violation of their confidentiality, unconsented testing and disclosure of HIV status, and verbal abuse [10, 13–16], while seeking care. The detrimental effects of stigma on the health outcomes of PLHIV are well documented [17], such as higher risk-taking behaviour [18, 19], refusal to disclose the HIV status [20], low uptake of HIV testing, care and support services [21–24], and poor adherence to treatment [25–27]. Stigma also undermines the mental health and quality of life PLHIV [19, 28]. These concerns are the rationale for the increased efforts to combat the HIV stigma in general and specifically in health settings [29].

In Yemen, one of the least developed countries [30], HIV prevalence remains low at 4000 (2000-11,000) per 100,000), with a possible epidemic concentrated among men who have sex with men (MSM) [31]. Estimates from programmatic data indicate a total of 30,000 PLHIV, most of whom reside in Sana’a City (35%) [32]. HIV diagnosis and treatment services are provided in designated centres under the management of the National AIDS Control Program (NAP). The NAP relies on external funding, which has gradually declined following the global prioritization of the high HIV-prevalence areas [33], and the need to encourage domestic funding of HIV control [34].

The outbreak of a violent internal conflict in March, 2015 and external air strikes campaign by the Saudi-led Coalition created one of the world’s largest humanitarian social and economic crisis [30]. The national health system has been eroded in terms of financing, human resources and infrastructure [35]. Only 8.5% of the already underfunded humanitarian support is directed to health, mainly primary health care (PHC), and child and maternal health programs [36]. Less than 50% of public health facilities are considered fully functional, and staff continue to work with shortages of medicines, supplies and equipment, and sporadic payment of salaries [37].

The NAP and humanitarian agencies continue to struggle to maintain access to as many PLHIV as possible. The services are extremely inadequate, and funds have to be diverted from the preventive and support aspects to sustain the testing and treatment services [38]. Many PLHIV have lost access to HIV care services, due to lack of personal financial resources, the closure of several NAP centres, increased transport costs, and road closures for security reasons [39]. PLHIV usually attend public health facilities for general ailments. They tend to seek care only if necessary, but conceal their HIV status when doing so out of fear of stigma [39]. One study reported that 56% of the health care providers in the public hospitals in Sana’a City practised HIV stigma, such as refusal of treatment and irrational use of personal protection measures [40]. Research about HIV stigma and HIV in general is very limited in Yemen. In view of this context, a better understanding HIV stigma in the health facilities is required to reduce its prevalence and thereby provide equitable and accessible health care services for PLHIV and populations at risk [29]. The aim of this study was to determine the prevalence and drivers of HIV stigma among the Yemeni health professionals in the teaching hospitals in Sana’a City, and its relationship to the current conflict and low-resource context of the country.

## Methods

### Study design and setting

This was a cross-sectional hospital-based study at the four teaching hospitals in Sana’a City, namely, Al-Thawrah, Al-Jomhouri, Al-Kuwait, and Al-Sabeen. The study hospitals serve populations from different parts of the country as referral and teaching facilities. The hospitals, though tertiary, receive self-referred patients with a range of ailments in the outpatient departments. Sana’a City is within average coverage in relation to health care services and health care providers [39].

### Sample size and sampling technique

The study included 320 Yemeni health professionals from the General Surgical, Obstetrics and Gynaecology, and Dentistry departments, where staff are likely to have contact with body fluid invasive interventions. The sample was calculated using Epi Info, Version 3.01, and based on a total population of 1651 registered providers of health care 95% confidence level. Due to the lack of updated estimates in Yemen, the stigma prevalence was set at 50% so as to obtain the largest sample size possible to capture the variance of binomial distribution of the stigma practices. The variance of the binomial distribution = *n* × p × (1 - p), which has a maximum at  p= 50% for any given *n*, where *n* refers to the size of the study population, and p is the prevalence of the outcome variable. The sample was distributed proportionally to the number and types of health professionals in the hospitals (Table 1). We approached all the staff who were present in the outpatient departments and wards until the sample size was achieved. There were no refusals to answer.

**Table 1:** Characteristics of the study participants

Attribute (***n*** = 320)	Frequency	Percentage
**Hospital**		
Al-Thawrah	211	66
Al-Jumhouri	51	16
Al-Sabeen	37	11.5
Al-Kuwait	21	6.5
**Specialization**		
Surgeons	16	5
Obstetricians and Gynaecologists	21	6
Dentists	14	4
Nurses	269	84
**Sex**		
Male	219	68
Female	101	32
**Age group (years)**		
20–29	159	50
30–39	113	35
40–49	18	5.5
50 or more	6	2
Missing	24	7.5
**Highest level of education**		
Nursing Diploma	222	69
Bachelor’s degree	85	27
Arab Board of Specialization (Medical Doctorate)	7	2
MSc	3	1
PhD	3	1
**Specialisation**		
General surgeons	16	5
Gynaecologist& Obstetrician	21	7
Dentists	14	4
Nurses	269	84
**Years of experience**		
< 2 years	50	16
2–5 years	99	31
6–10 years	78	24
> 10 years	93	29

### Study variables and tool

A paper-and-pencil self-completed questionnaire was modified from the Health Policy Project Tool (HPPT) [41]. The HPPT measures HIV stigma and its drivers. It was formulated by a group of PLHIV and a spectrum of professionals working in HIV through a multi-stage process of content development. The resulting questionnaire was field tested in health facilities at six diverse sites. The final HPPT included 18 core questions measuring 3 programmatically actionable drivers of stigma (worry about HIV transmission, attitudes towards PLHIV, and health facility environment, including policies), and enacted stigma. The questionnaire also included a short scale for attitudes towards PLHIV (5-item scale, *α =* 0.78) [41]. In the current study, the tool was pilot tested among 5 health care providers and modified accordingly. The final questionnaire was arranged as follows: I. Background information: demographic, education, and job duties (6Q); II. Drivers of stigma: previous HIV training, contact with PLHIV (6Q), fear of infection (1Q with 4 sub-items), attitudes towards PLHIV (8 Q), willingness to treat key population (1 Q with 3 sub-items), and health facility environment in terms of adequacy of infection control measures and observed stigma (3 Q); III. Enacted stigma: irrational use of infection control measures (1Q with 4 sub-items), unconsented HIV testing (1Q), breach of confidentiality (Q3), and secondary stigma (1Q with 4 sub-items).

### Data collection

Data were collected by a team of trained medical students in October 2016. Health professionals working in the outpatient departments and in the wards were approached and asked to participate in the study. Questionnaires were handed out to the participants and were collected anonymously afterwards.

### Data analysis

Descriptive statistics were used to analyse the different variables. Cross tabulation and Pearson correlation were used to determine how the providers’ characteristics and the potential drivers of stigma relate to the reported individual stigmatizing practices. It was not feasible to sum these practices into one variable (Stigma/No stigma) because all participants had committed at least one form of enacted stigma. Chi square was calculated, and results were considered statistically significant where the *p *value was equal to or less than 0.05. We used the SPSS program version 22 (NY, USA) [42].

### Ethical consideration

The study was approved by the Faculty of Medicine and Health Sciences, Sana’a University. Informed consent was obtained verbally from the participants prior to handing out the questionnaire. The participants were able to refuse participation, withdraw at any point or ignore any question. They were assured of the anonymity and confidentiality of the information. No personal identifiers were included in the questionnaire and completed forms were collected in sealed unmarked envelopes.

## Results

### Background information

Table 1 shows the personal and professional characteristics of the participants. Half of the participants were in the 20–29 years age group and just over a third were 30–39 years old. The majority of the sample were females (68%), and nurses from the different departments (84%). Regarding the level of education of participants, the majority had a Nursing Diploma (68%) or Bachelor’s degree (27%).

### Enacted stigma

The majority of the 320 participants said they would avoid physical contact with patients living with HIV (87%), wear gloves throughout the consultation (96.5%), wear double gloves (91%), or destroy the instruments used in care provision (80%). In addition, most of the providers felt they had the right to disclose the HIV positive status to the spouse (93%), relatives (88%), and to colleagues (99%). Table 2 shows that a considerable proportion of the providers applied at least one form of unnecessary infection control, and almost all would breach patients’ confidentiality (99%) during provision of care for PLHIV.

**Table 2:** Prevalence of enacted HIV stigma

Statement	Always	Often	Sometimes	Seldom	Never
**While providing care to PLHIV, I: (*n*** **= 320)**					
Avoid physical contact with PLHIV (N:310)	175 (55%)	39 (12%)	34 (11%)	30 (9%)	42 (13%)
Wear gloves during provision of all aspects of care	254 (79.5%)	32 (10%)	14 (4%)	9 (3%)	11 (3.50%)
Wear double gloves	178 (56%)	54 (17%)	33 (10%)	26 (8%)	29 (9%)
Destroy the instruments I used in providing the care	148 (46%)	31 (10%)	36 (11%)	41 (13%)	64 (20%)
**Breaching confidentiality (*n*** **= 320)**					
I have the right to disclose the HIV status to the spouse	232 (73%)	42 (13%)	20 (6%)	3 (1%)	23 (7%)
I have the right to disclose to relatives	192 (60%)	34 (10.5%)	37 (11.5%)	19 (6%)	38 (12%)
I have the right to disclose to another health care provider	286 (89%)	16 (5%)	10 (3%)	5 (2%)	3 (1%)

### Potential drivers of HIV stigma

Stigma drivers include professionals’ HIV knowledge and training, worry about HIV infection while providing care, attitudes towards PLHIV, preference to treat key populations, and facility environment and policies.

#### Professionals’ HIV knowledge and training, and contact with PLHIV

**Fig. 1:**
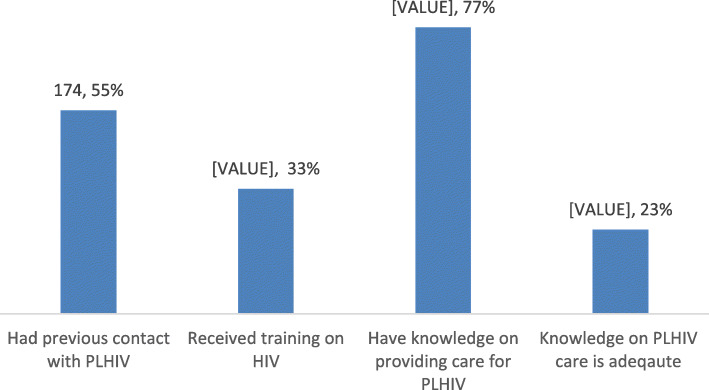
Participants’ level of HIV knowledge, HIV training and contact with PLHIV (*n*, %)

Figure 1 shows that only 33% of the participants had received any form of training on HIV, although 77% mentioned that they had knowledge on how to provide care for PLHIV appropriately. About 23% of participants were confident that their knowledge was adequate. This was also evident from responses about the mode of transmission of HIV, which showed that 123 out of 320 participants (38%) wrongly mentioned that HIV could be transmitted through tears, sweat and urine. More than half of the respondents had previous contact with PLHIV (55%), and for half of these respondents, this contact was with patients in the health facilities (Fig. 1).

#### Fear of infection

Many participants were worried about acquiring HIV infection while providing care for PLHIV. The higher the invasiveness of the procedure performed, the larger was the proportion of the providers who expressed worry. Table 3 shows that 59% of the participants were worried when they measured body temperature, whereas 79% were concerned about physical contact with the patient, and 88% when they drew blood sample or bandaged a wound for a patient living with HIV.

**Table 3:** Potential drivers of HIV stigma

Fear of HIV infection (***n*** = 320)	Very much	Much	A little	Too little	Not worried
**The extent of a professional’s worry when providing care for PLHIV while:**					
Measuring temperature	25 (8%)	27 (8%)	92 (29%)	52 (16%)	124 (39%)
Touching personal things	87 (27%)	53 (17%)	78 (24%)	36 (11%)	66 (21%)
Bandaging a wound	138 (43%)	50 (16%	54 (17%	39 (12%)	39 (12%)
Drawing blood sample	141 (44%)	59 (18%	50 (16%)	32 (10%)	38 (12%)
**Stigmatizing attitudes towards PLHIV**	**Strongly agree**	**Agree**	**I don’t know**	**Disagree**	**Strongly disagree**
HIV is a punishment (*n* = 315)	23 (7%)	18 (6%)	36 (11%)	116 (37%)	122 (39%)
PLHIV are responsible for their infection (*n* = 318)	49 (15.5%)	61 (19%)	33 (10.5%)	120 (38%)	55 (17%)
Most PLHIV do not care if they infect other people (*n* = 312)	23 (7%)	71 (23%	120 (38%)	65 (21%)	33 (11%)
Women living with HIV should not be allowed to have babies. (*n* = 313)	104 (33%)	64 (20.5%)	42 (13.5%)	71 (23%)	32 (10%)
Patients should be tested for HIV prior to surgical procedures even without prior knowledge or consent (*n* = 316)	174 (55%)	70 (22%)	12 (4%)	47 (15%)	13 (4%)
HIV services should be provided in separate centres	173 (54%)	82 (25.5%)	18 (5.5%)	41 (13%)	6 (2%)
It is better to avoid working alongside a co-worker living with HIV (*n* = 314)	28 (9%)	52 (16.5%)	34 (11%)	146 (46.5%)	54 (17%)
PLHIV should not work in health care provision	83 (26%)	66 (21%)	33 (10%)	96 (30%)	42 (13%)
**Health facility environment and policies**	**Strongly agree**	**Agree**	**I don’t know**	**Disagree**	**Strongly disagree**
There are adequate supplies in my health facility that reduce my risk of becoming infected with HIV	6 (2%)	15 (4%)	25 (8%)	102 (32%)	172 (54%)
The international HIV infection control guidelines are not adequate	141 (44%)	97 (30%)	60 (19%)	14 (4.5%)	8 (2.5%)
	**Yes**	**Not sure**		**No**	
I will get in trouble at work if I discriminate against patients living with HIV. (*n* = 310)	170 (55%)	62 (20%)		78 (25%)	

#### Attitudes towards PLHIV

Providers’ responses varied in this category producing a different number of answers in the attitude items. As shown in Tables 3, 13% agreed that HIV infection is a “God’s punishment” for their “bad” behaviour. In addition, higher proportions of providers agreed with the statements: most PLHIV do not care if they infect others (30%); PLHIV are responsible for their infection (35%); women living with HIV should not be allowed to get married or have babies (54%). Within the context of health facilities, 77% of the respondents agreed with the statement that patients should be tested for HIV prior to surgical procedures, even without their prior knowledge or consent, and 79% agreed that HIV services should be provided in separate centres. A quarter of the respondents mentioned that it is better to avoid working with PLHIV, whereas 47% agreed that health workers living with HIV should stop working in health care provision.

#### Preference to treat key population

When asked about their attitude regarding providing treatment to key populations, 62% of the participants preferred not to treat persons who inject drugs. This proportion was substantially higher than that for those who preferred not to provide treatment to MSM (34%) or sex workers (33%). Please, note that data are not included in the tables.

#### Health facility environment

None of the hospitals had specific policies or procedures against HIV stigma. Regarding infection control measures, only 7% the providers believed that the Personal Protective Equipment (PPE) and supplies were adequate (Table 3). In terms of observed stigma, 36% of the practitioners witnessed acts of stigma by a colleague towards a patient living with HIV. The most commonly reported forms were: unconsented disclosure of a patient’s HIV status (*n* = 93, 82%); the irrational use of infection control measures (*n* = 73, 64%), avoiding the patient and unjustified referral (*n* = 69, 60.5%) and carelessness while providing care (*n* = 61, 53.5%). In comparison, 12% (*n* = 14) witnessed PLHIV being subjected to verbal or physical abuse. Please, note that data pertaining to observed stigma are not included in the tables. Finally, 55% of the participants indicated that discrimination against PLHIV would lead to repercussions at work, 20% were not sure, but 25% did not think so.

### Drivers of HIV stigma

The correlations between the different study variables are presented in Supplementary File 1. Avoiding physical contact with PLHIV was moderately but significantly correlated with fear of HIV infection after touching the patients (0.190, *p* < 0.01), while drawing a blood sample (0.150, *p* < 0.01), and during measuring body temperature (0.240, *p* < 0.01), and also inversely correlated with the participants’ perception of their HIV knowledge as adequate (0.206, *p* < 0.01). The use of gloves throughout the clinical session was strongly correlated with fear of infection during wound dressing (0.675, *p* < 0.01), whereas using double gloves was significantly correlated with specialization (0.150, *p* < 0.01), and holding negative opinions towards PLHIV (0.163, *p* < 0.01). Destroying instruments was correlated with fear of infection on measuring the temperature (0.133, *p* < 0.05) and holding negative attitudes (0.179, *p* < 0.01). There were weak although significant correlations between disclosing the HIV status to a relative and fear of HIV infection while measuring the body temperature (0.120, *p* < 0.05), and holding negative attitudes towards PLHIV (0.125, *p* < 0.05). Perceived adequate knowledge was significantly correlated with having had HIV training (0.208, *p* < 0.01), and having previous contact with PLHIV whether in the family (0.213, *p* < 0 .01) or in the community and work (both 0.177 and *p* < 0 .01). Receiving specific HIV training was inversely and significantly associated with the fear of infection on drawing blood samples from PLHIV (0.120, *p* < 0.05).

**Fig. 2:**
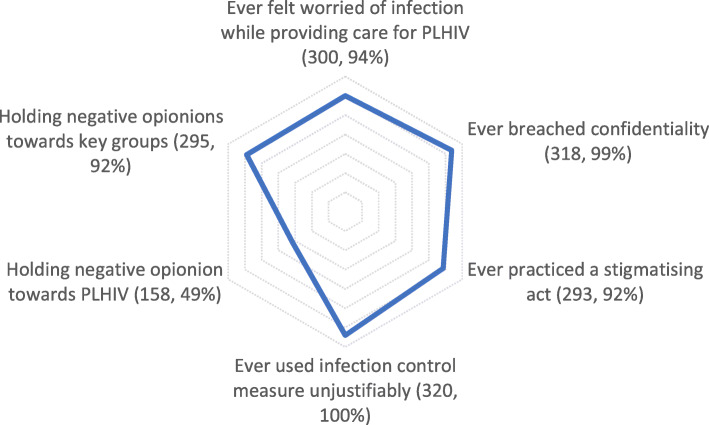
Overview of enacted stigma and its drivers (frequency, percentage)

Figure 2 summarises the study results regarding HIV stigma and its drivers in the teaching hospitals in Sana’a City. It shows that all the professionals in our study would use infection control unjustifiably when providing care for a person living with HIV, 99% would breach the patient’s confidentiality, and 94% feel worried when providing the care for PLHIV. In contrast, 49% hold negative views against PLHIV.

## Discussion

This was a cross-sectional study that examined HIV stigma among 320 Yemeni health professionals working in departments with high body fluid exposure in four teaching hospitals in Sana’a City, Yemen. The results showed a high level of HIV stigmatizing attitudes and practices in these teaching hospitals. Irrational use of infection control measures and unconsented disclosure of the patients’ HIV status to other practitioners were almost universal among health professionals. The majority of participants felt that HIV testing should be done before surgical interventions even without prior knowledge or consent, and HIV services should be provided in separate centres. Comparatively high level of stigma were reported in urban India; almost 90% of doctors and nurses stated that they would discriminate against PLHIV in professional situations that involved high likelihood of fluid exposure [10]. In Saudi Arabia, a study among senior dentistry students showed that 90% would use an extra pair of gloves, and 95% referred patients living with HIV to a specialist [43]. Lower levels of stigma have been reported in other settings [44–47]. For example, half of the nurses and doctors in Lao have a high level of stigma against PLHIV [47], and among health care providers in Ghana, 35% were unwilling to treat PLHIV and 52% would use unnecessary protection covers when seeing at a patient (thought to be) living with HIV [44]. If given the choice, about 40% of the doctors in Saudi would not work with PLHIV [48].

HIV stigma in our study was significantly associated with the participants’ fear of infection, perceived poor HIV knowledge, and negative attitudes towards PLHIV. These findings are consistent with reports that identify fear of infection as a key driver of HIV stigma, fuelled by a perception of high risk of infection [10–12, 14, 49, 50]. Similarly, poor knowledge of HIV transmission and prevention is linked to stigma [49, 51, 52], whether directly [53], or as a mediator of fear of infection [54, 55]. Similar to other settings, the social component still plays a role in Yemen as indicated by the significant association between stigma and the professionals’ negative attitudes towards PLHIV [54, 56–58].

Contextual factors play an important role in our study, an aspect that is inadequately addressed in HIV stigma research [54]. Our findings corroborate the notion that HIV stigma in low-HIV prevalence and low-resource communities [59, 60]. Funding for HIV programs is usually scarce, professionals’ contact with PLHIV is uncommon [23, 61], and HIV knowledge is poor in these contexts [51, 54]. HIV stigma indicators among professionals in Sana’a teaching hospitals have deteriorated following years of conflict. Between 2010 [40] and 2017 (the current study), HIV stigma prevalence rose from 56% to almost 100%. In addition, poor HIV knowledge increased from 12 to 38%, reported PPE shortage rose from 69 to 93%, and the perception of risk of infection soared from 13% to 94% among health professionals. Moreover, the current study shows that the stigma is associated with poor HIV knowledge and fear of infection. In contrast, the professionals’ negative attitudes towards PLHIV, not HIV knowledge or fear, predicted the stigma in 2010 [40], which suggests a shift from a social-based towards a fear-based HIV stigma. These findings call for a comprehensive multi-level strategy to address the stigma and its drivers in Sana’a hospitals, building on the growing global experience in combating HIV-related stigma [62–64].

At the institutional level, antidiscrimination policies should be developed, shared with the health workers, and effectively applied. Structural adjustments are required, such as improved availability of hand-washing facilities and ensuring the availability of sharps containers and PPE. At the individual level, training is needed on HIV and universal precautions. Negative attitudes towards PLHIV should be tackled by, for example, embedding testimonials from PLHIV in the staff training workshops and ensuring PLHIV participation in the development of hospital policies.

The implementation of these interventions is challenging due to lack of funds. As previously stated, the NAP is excluded from humanitarian funding and is incapable of playing its role in controlling HIV or combating the related stigma. The UN has declared its commitment to control HIV and combat stigma during conflict as well as peace [65]. Despite the high UN level commitment, most of the limited health funds are directed to the PHC and maternal and child health programs [36]. The focus on the latter programs and subpopulations, although vulnerable, overlooks the needs of other vulnerable groups such as the PLHIV, and risks undermining the HIV control efforts. Therefore, funds need to be reallocated to ensure the required financial resources support the health facilities rather than certain type of services, and thereby meet the needs of a wider spectrum of the community. Joint programming should also be encouraged so as to efficiently use the limited resources e.g., mainstream the HIV knowledge and stigma education interventions in the undergraduate teaching programmes and in ongoing in-service training sessions.

There are a number of limitations that should be considered in interpreting the results. Firstly, the study was conducted in the hospital departments with high fluid exposure, which may increase the perceived risk of infection and in turn stigma. In addition, the results may not be generalizable to other levels of health care because of differences in the available resources, type of health care provided, and the health professionals’ qualification and training. There may be concern about response bias, where the providers would record what they are expected to do rather than what they practise and thereby underestimate the level of stigma, but the responses indicate the contrary. This might be explained by the fact that the data were collected confidentially, or that the professionals considered these practices acceptable. Finally, the study tool was developed for operational research purposes and we were not able to construct measurement scales or establish its measurement properties. Despite these limitations, this study was able to reveal the widespread stigma in the teaching health facilities and possible links to the context of low HIV prevalence, low resources, and conflict. Our study provides useful data to help design and evaluate HIV control interventions in hospitals.

## Conclusions

There is widespread HIV stigma in the major hospitals in Yemen, consistent with previous reports of high stigma in low HIV prevalence and low-resource countries, and its association with the fear of infection, poor HIV knowledge, inadequate training, and limited allocation of funds to HIV control programmes. Notwithstanding the current financial limitations of HIV control in Yemen, stigma reduction interventions need to be mainstreamed at institutional and individual levels. In addition, anti-discrimination policies and structural adjustments should be implemented, in combination with training on HIV and universal precautions, and action taken to tackle negative attitudes towards PLHIV and key populations.

## Supplementary Information

**Additional file 1.** The master table of correlations between the participants’ personal and professional characteristics, attitudes towards key populations on one hand, and the individual stigma practices on the other.

## Data Availability

Dataset generated and analysed for the current study is available from the corresponding author on reasonable request.

## Abbreviations

NAP: National AIDS Programme
PHC: Primary Health Care
PLHIV: People Living with HIV
PPE: Personal Protective Equipment
WHO: World Health Organization
